# BMC ecology image competition 2018: the winning images

**DOI:** 10.1186/s12898-019-0226-z

**Published:** 2019-03-08

**Authors:** Alison L. Cuff, Ying Lou, Jiang Zhigang, Michel Baguette, Simon Blanchet, Jean Clobert, Luke M. Jacobus, Dominique Mazzi, Josef Settele

**Affiliations:** 1 BMC, Berlin, Germany; 2 BMC, Shanghai, China; 3 University of Chinese Academy of Sciences, Beijing, China; 4 National Museum of Natural History, Paris, France; 5 Laboratoire d’Ecologie Expérimentale du CNRS à Moulis, Saint-Girons, France; 6 Indiana University-Purdue University Columbus (IUPUS), Columbus, Indiana 47203 USA; 7 Agroscope, Müller-Thurgau-Strasse 29, 8820 Wädenswil, Switzerland; 8 Helmholtz Centre for Environmental Research–UFZ, Leipzig, Germany

## Abstract

The sixth *BMC Ecology* Image Competition received more than 145 photographs from talented ecologists around the world, showcasing the amazing biodiversity, natural beauty and biological interactions found in nature. In this editorial, we showcase the winning images, as selected by our guest judge, Professor Zhigang Jiang from the Institute of Zoology of the Chinese Academy of Sciences, with help from the journal’s editorial board. Enjoy!

**Electronic supplementary material:**

The online version of this article (10.1186/s12898-019-0226-z) contains supplementary material, which is available to authorized users.

## Editorial

The *BMC Ecology* Image Competition gives ecologists the opportunity to present their research in a visual and entertaining way, using photography to draw attention to some of the outstanding research that is being conducted in the field. Our past competitions captured many stunning aspects of ecologists’ view of the world [1–5], and we were eager to continue and build on our past successes in 2018.

We were delighted that Professor Jiang Zhigang agreed to be our guest judge. Professor Jiang is not only a senior ecological scientist from the Institute of Zoology of the Chinese Academy of Sciences, but also a photographer. During his more than 40 years of wild animal and ecology research he has traveled all over China and visited dozens of countries and regions, using his camera to record the focus of his research and the environments in which they can be found His expertise and experience as a scientist and photographer made him an excellent judge for the 2018 *BMC Ecology* image competition. As in previous years, the editorial board of *BMC Ecology* provided their support as judges, to select the winners for each of the five section categories. All judges were unaware of the photographers’ identities and selected the winners based solely on the evaluation of the photo. These impressive images received for the 2018 competition gives us a better understanding of global diversity—not only from the prospective of in nature and ecology, but also from the people working in the field. We hope that you will enjoy experiencing some of the wonders of nature and learn something unexpected from this visual feast.

## Winning images

**Fig. 1:**
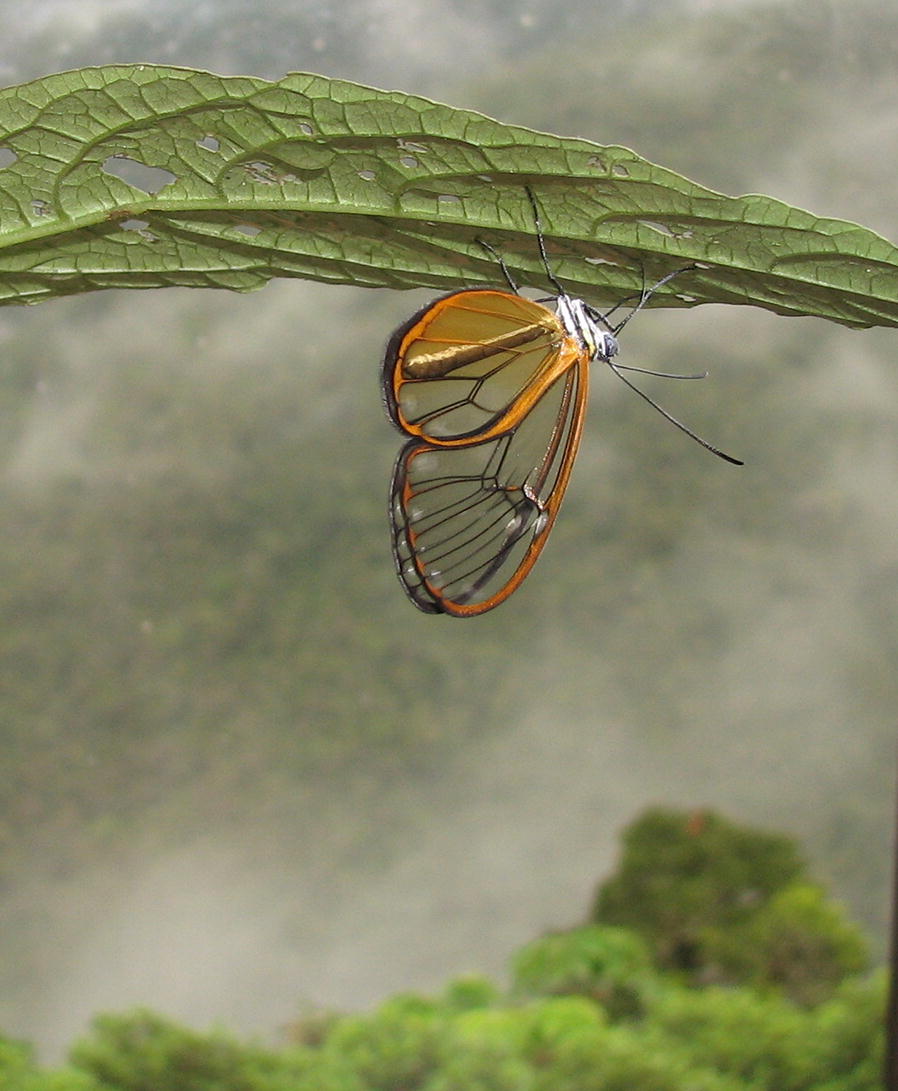
Overall winner: the enigmatic clearwing butterfly *Hypomenitis enigma* (Nymphalidae:Ithomiini). This photo was taken in the southern Andes of Ecuador (2000 masl, Zamora-Chinchipe province) 2 h after the butterfly had emerged from its chrysalis Attribution: Marianne Elias (Institut de Systématique, Evolution, Biodiversité, CNRS, MNHN, Sorbonne Université, France)

Our overall winning image of the clearwing butterfly *Hypomenitis enigma*), was taken by Marianne Elias of Sorbonne University, France (Fig. 1).

Marianne Elias said: “The clearwing butterfly inhabits cloud forests in the Andean mountains. Wing transparency is caused by dramatic modifications to the shape of its wing scales (which look like hair), and by the presence of tiny structures on the wing surface that act as anti-reflectors, thereby increasing the amount of light transmitted through the wing. Evolution of transparency in this species and its allies raises multiple questions. How can transparent wings with reduced membrane coverage by scales provide optimal hydrophobicity and thermoregulation in the moist and relatively cold habitats occupied by *H. enigma*? What ecological role does transparency, a feature commonly associated with camouflage play in *H. enigma* and its allies? These questions are currently being explored by an international and interdisciplinary team of researchers. This photograph was taken in the southern Andes of Ecuador (2000 masl, Zamora-Chinchipe province) 2 h after the butterfly had emerged from its chrysalis.”

Our “Behavioral and physiological ecology” section Editor Dominique Mazzi also admired this image. “This photo does a fantastic job of showcasing how the rare butterfly’s beauty evolved from the environment where it lives. It is a worthy winner of this year’s competition”.

## Runners up

**Fig. 2:**
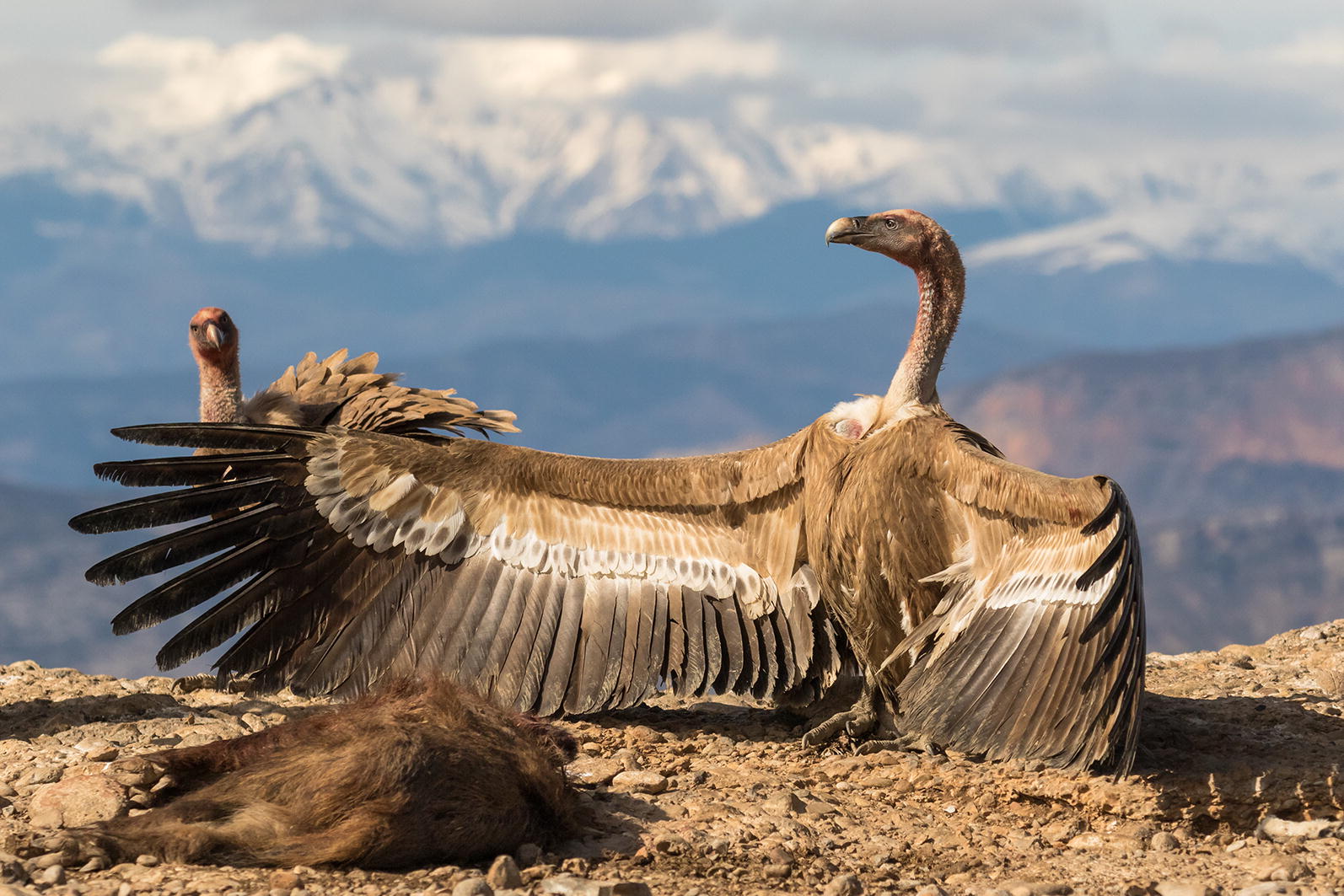
Runner-up: my treasure! Attribution: Pilar Oliva Vidal (University of Lleida—ETSEA, Spain)

Our first runner up is an image showing the dominance behavior of the griffon vulture (Fig. 2). Pilar Oliva Vidal from University of Lleida, Spain captured this wonderful moment, titled ‘My treasure!’

As Vidal explains, “The griffon vulture in the photo shows dominance behavior, shielding a carcass of a dead wild boar, to prevent the approach of others. Vultures play a hugely important role in the ecosystems they inhabit, because they clean up carcasses very efficiently. They can detect and strip a carcass in a few minutes—which inhibits the spread of pathogens and reduces the need for the treatment and incineration of domestic animal remains, as well as avoiding the resulting emissions of thousands of tons of carbon dioxide.”

**Fig. 3:**
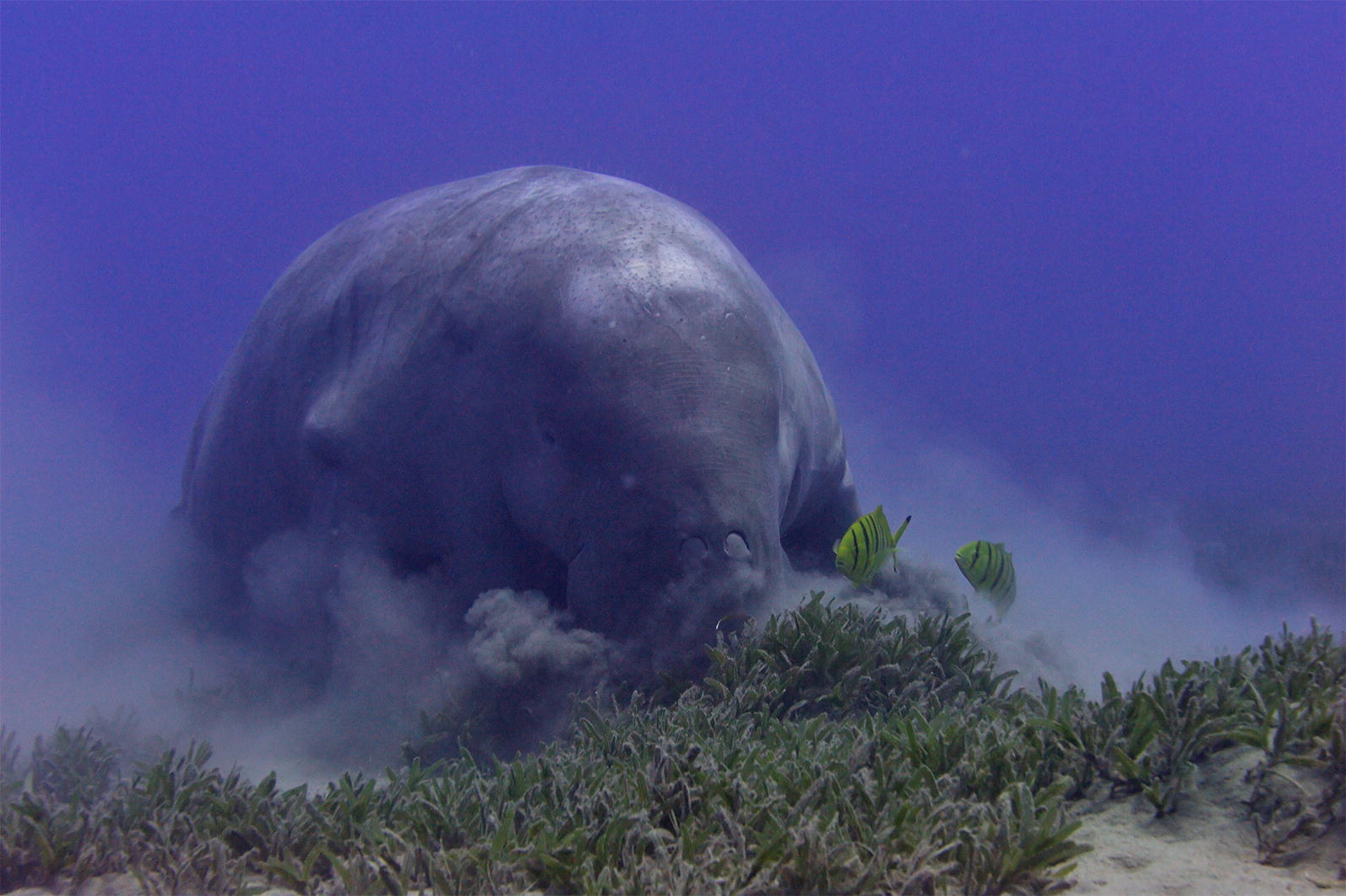
Runner-up: hungry dugongs have no table manners Attribution: Matteo Santon (University of Tuebingen, Germany)

Our second runner up is an image by Matteo Santon from the University of Tuebingen, Germany is entitled ‘Hungry dugongs have no table manners’ (Fig. 3).

When he was taking this picture, Dr. Santon did not use a flash, because it can affect the typical underwater short-wavelength skewed light spectrum (reds are almost absent below a sea depth of 10 m) by adding a lot of white light.

Santon describes the photograph: “This image shows a resident dugong foraging in one of the last large seagrass patches near Marsa Alam, Red Sea. Dugongs are extremely vulnerable to the loss of seagrass habitats, and are considered to be at risk of extinction. The dugong frenetically forages holding its breath at 24 m depth, while two pilot fish feed on its food scraps. The relationship between pilot fish and dugong is considered to be mutualistic: while the dugong offers the fish protection and sources of food, the pilot fish clean parasites off its skin.”

## Behavioral and physiological ecology

**Fig. 4:**
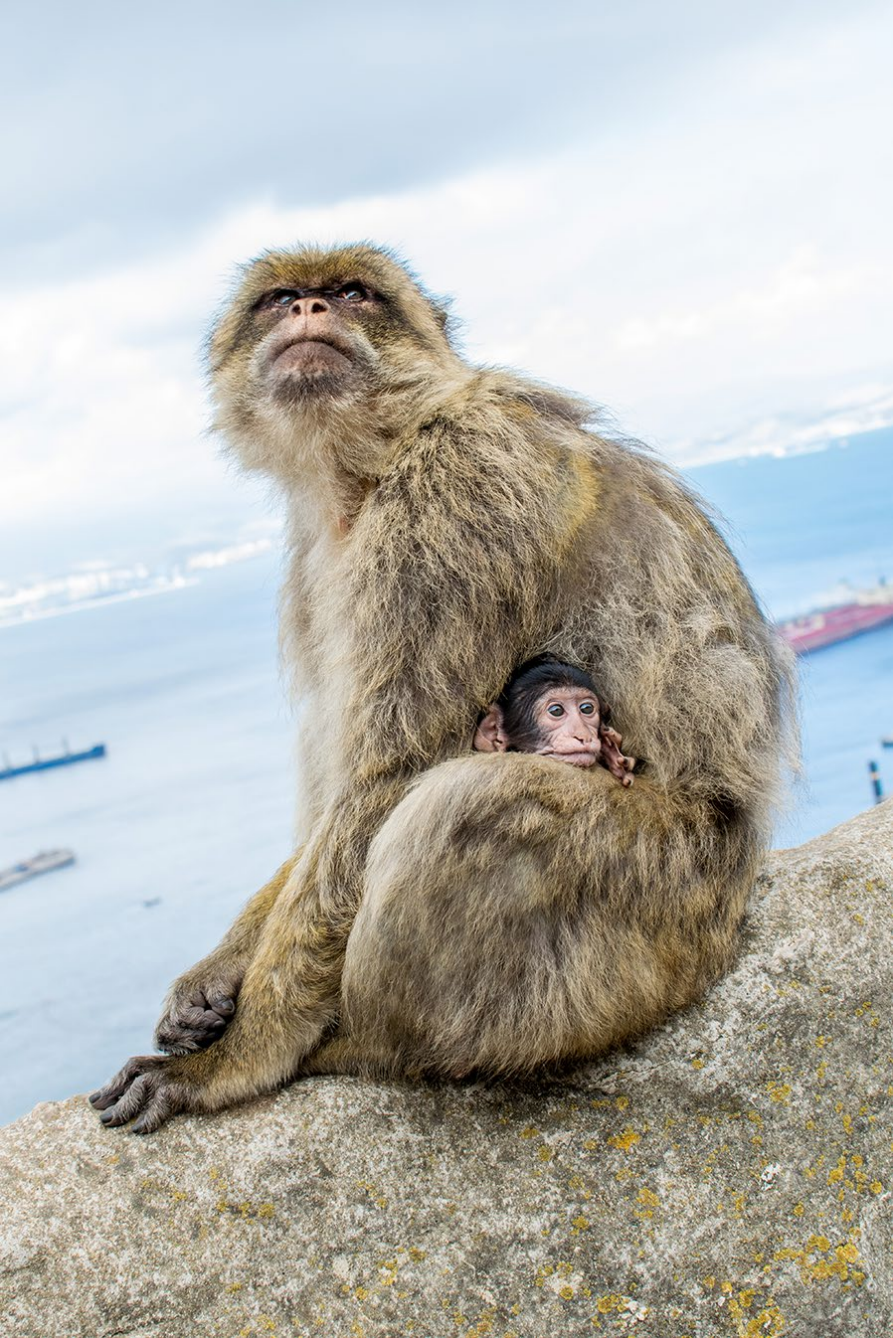
Winner: home in mother’s arms. Attribution: Arnaud Badiane (iEES-Paris, Sorbonne University, Paris, France)

Our winning image in this category was taken by Arnaud Badiane from France. ‘Home in mother’s arms’, and shows a young Barbary macaque (*Macaca Sylvanus*) tucked in close to its mother (Fig. 4).

Badiane explains: “For a young Barbary macaque (*Macaca Sylvanus*), home is not a place; home is the warmth only its mother’s arms can provide. This young macaque observes, through the window of its safe home, its fellow primates as they thrive on the rock of Gibraltar.”

## Conservation ecology and biodiversity research

**Fig. 5:**
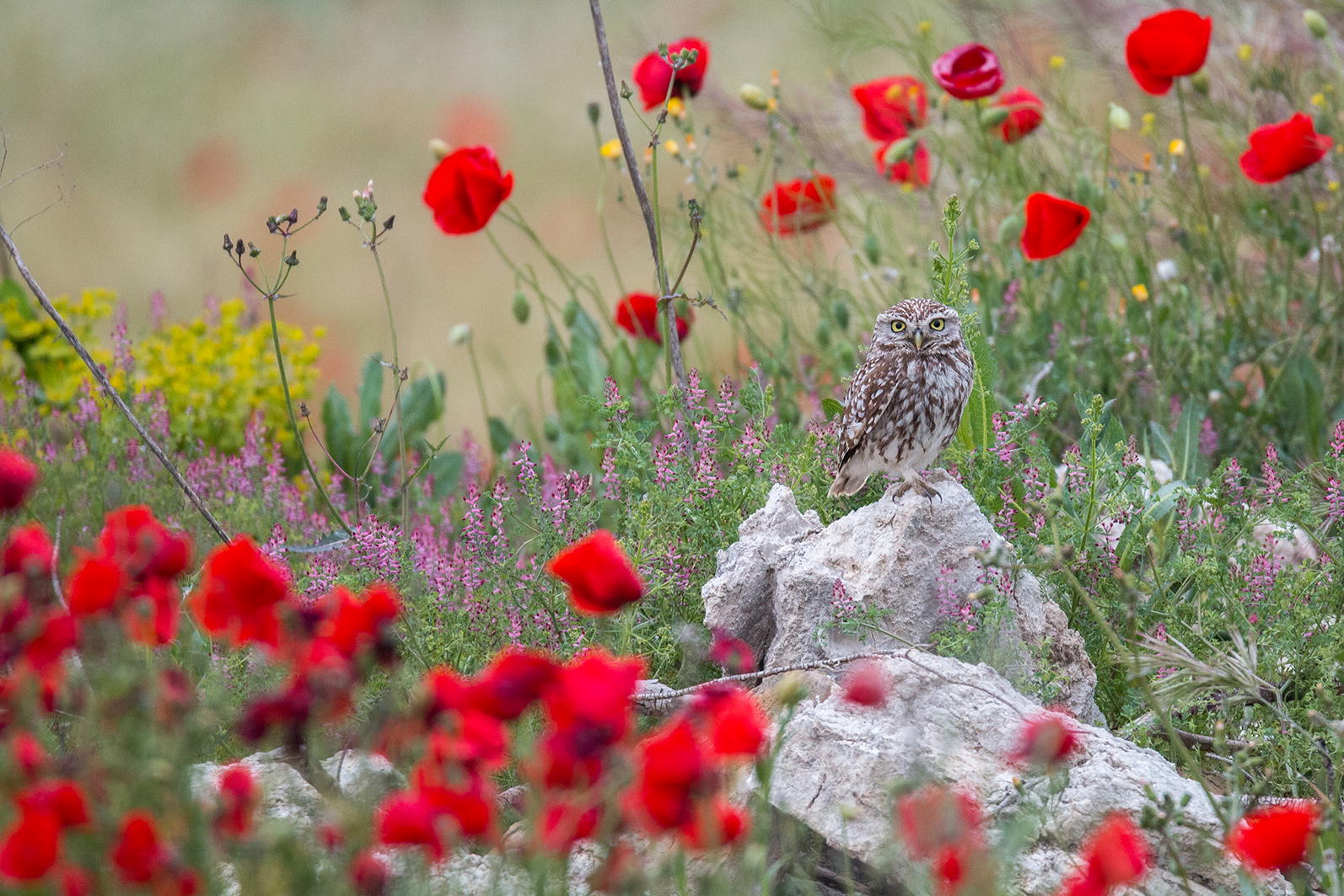
Winner: little treasures of the steppes. Attribution: Pilar Oliva Vidal (University of Lleida—ETSEA, Spain)

Chosen by Section Editors Luke Jacobus and Josef Settele, our winner in this category was another entry by Pilar Oliva Vidal, titled ‘Little treasures of the steppes’ (Fig. 5).

Josef Settele explained: “We chose this picture because of the composition of colors, and because it shows both the biotic and abiotic components of the featured ecosystem. We also very much liked the author’s story: on many occasions, steppe ecosystems are subject to human influence, mainly of intensive agriculture, which negatively affect flora and fauna. Steppe birds are amongst the most threatened groups of birds in Europe. They are very sensitive to the loss of steppe habitats. It is important to guarantee the conservation and recovery of these habitats and their species through the appropriate management of human activities.”

## Community, population and macroecology

**Fig. 6:**
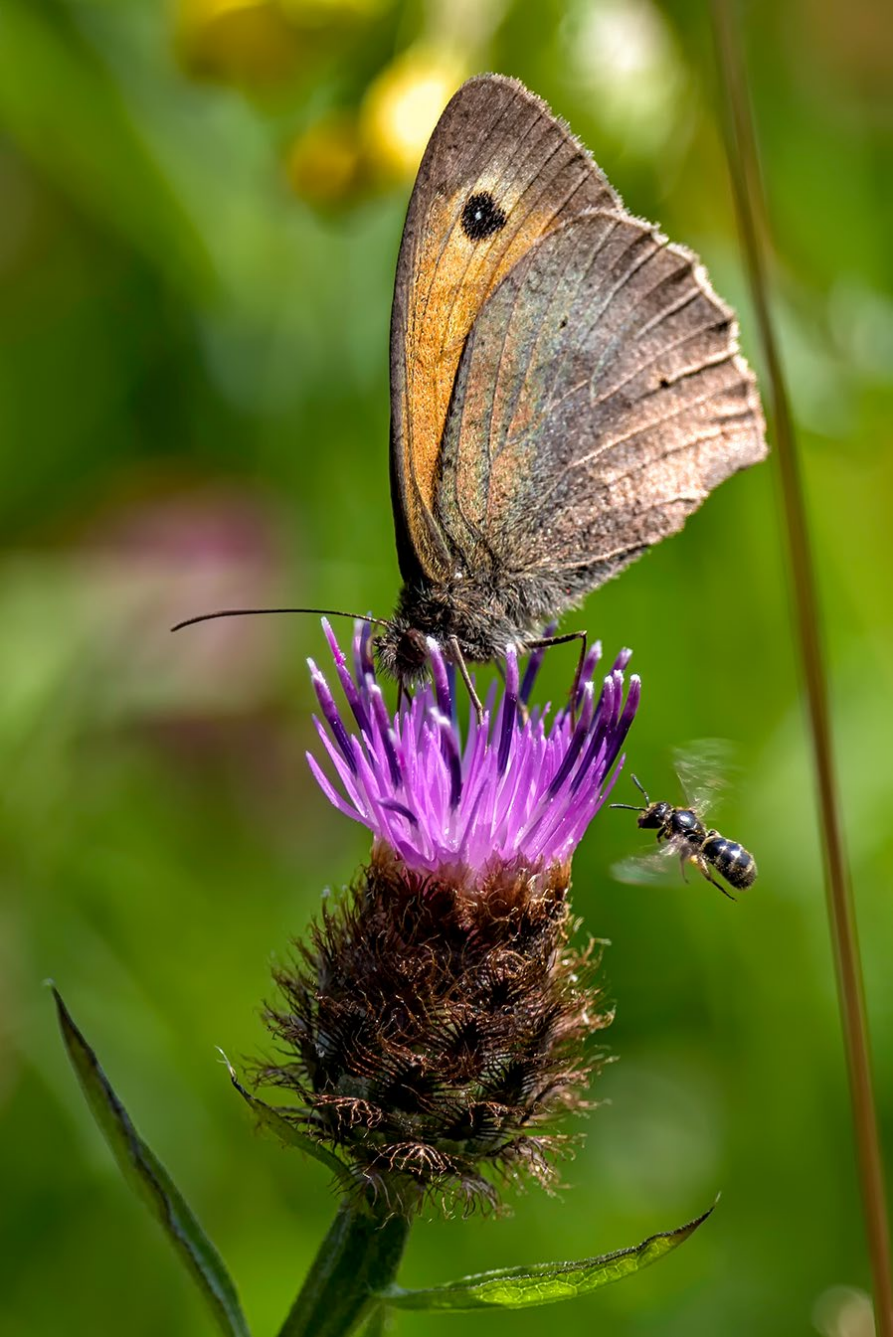
Section winner: Meadow Brown and solitary bee. Attribution: William Mills (University of Brighton, UK)

Our winner in this category is entitled ‘Meadow Brown and solitary bee’ (Fig. 6). The photo was taken by William Mills of University of Brighton, UK.

Mills describes the importance of the location where the photograph was taken and also his surprise at capturing the image of the bee. “Wildflower grassland habitat has greatly reduced in the UK over the last 80 years. However, due to its ability to support a wealth of flora and fauna, and particularly invertebrates, many local and district councils have been working hard to create and expand habitats within local and national nature reserves. One such reserve is the Bedelands Farm Local Nature Reserve in Burgess Hill, East Sussex. It was while I was volunteering on this reserve, working with the chairman Dominic Moore, that I stopped to take this photo of the male meadow brown (*Maniola jurtina*) feeding on a common knapweed (*Centaurea nigra*) flower. However, it wasn’t until I got home that I spotted the small solitary bee. The reserve has become one of my favorite places to visit with my camera.”

## Landscape ecology and ecosystems

**Fig. 7:**
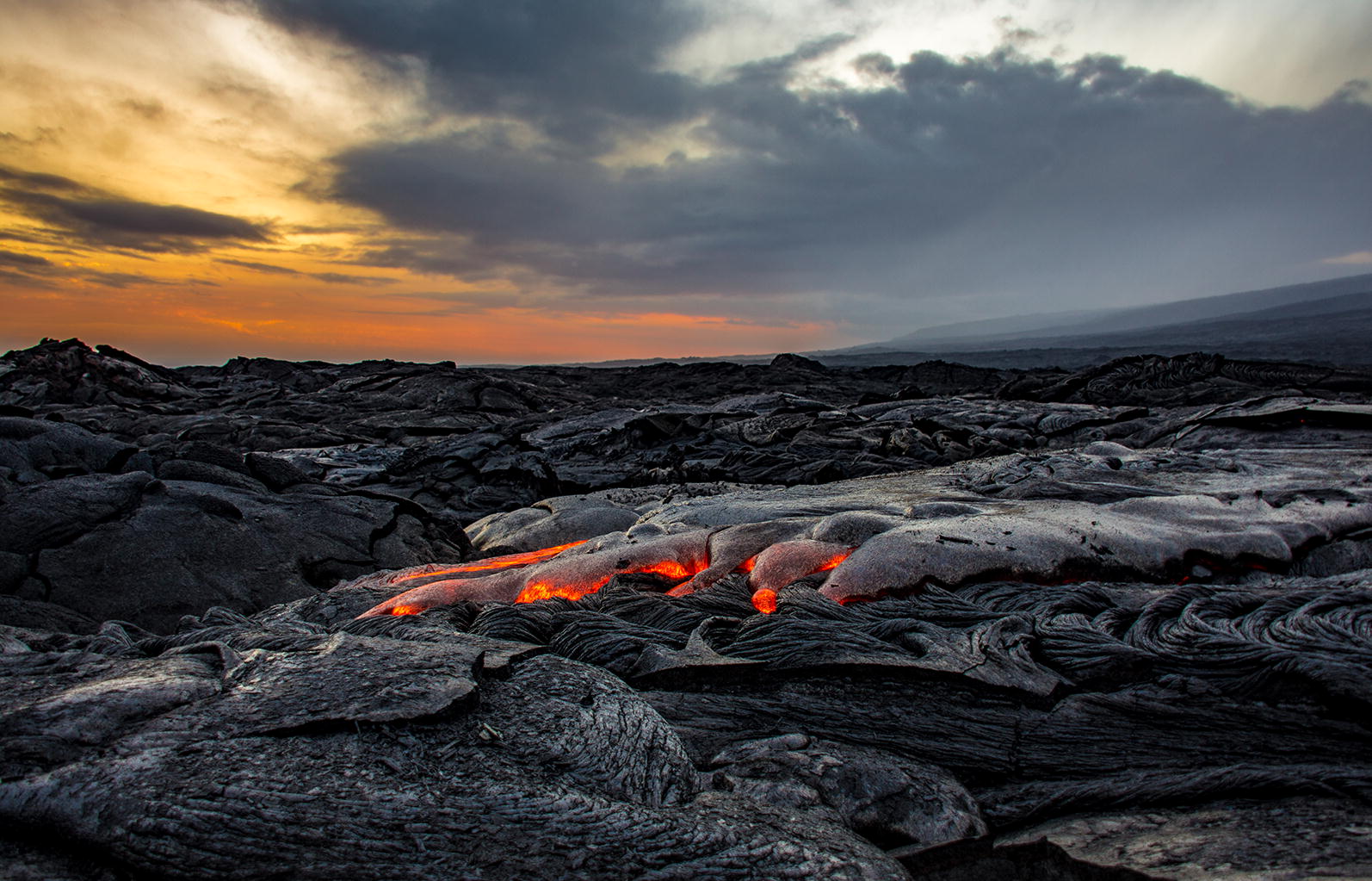
Section winner: Kilauea Volcano. Attribution: Sabrina Koehler (Kiel University, Germany)

This category’s winning image was taken on the island of Hawaii by Sabrina Koehler of Kiel University, Germany. It shows the Kilauea Volcano, captured (Fig. 7).

Dr. Koehler describes how she came to take the photograph: “During my postdoctoral study at the University of Hawai’i at Manoa, I visited the current Pu’u O’o eruption site of the Kilauea Crater (shown here at sunset). Hawai’i’s Big Island is still growing year on year and the recent lower Puna eruption of 2018 reminds us of the unpredictable nature of a volcano and its opposing forces of destruction and creation.”

Section Editor Michel Baguette explains his rationale for selecting this picture. “Landscapes can be defined as a patchwork of different habitats that develop according to local environmental conditions (climate, geomorphology) and disturbances. Ecological successions—the gradual processes by which ecosystems change and develop over time—drive habitats to their climactic states, while disturbances damage or destroy the climax and relaunch the successions. A landscape is thus a shifting mosaic in space and time of successional habitats generated by random or periodic disturbances. This picture is a nice illustration of the destructive power of an extreme disturbance that will create the opportunity of reinitiating ecological succession. It captures the simultaneous end and beginning of many eco-evolutionary processes—a wonderful example of what A. L. Lavoisier (1743–1792) famously stated: “nothing is lost, nothing is created, everything is transformed.”

## Editor’s pick

**Fig. 8:**
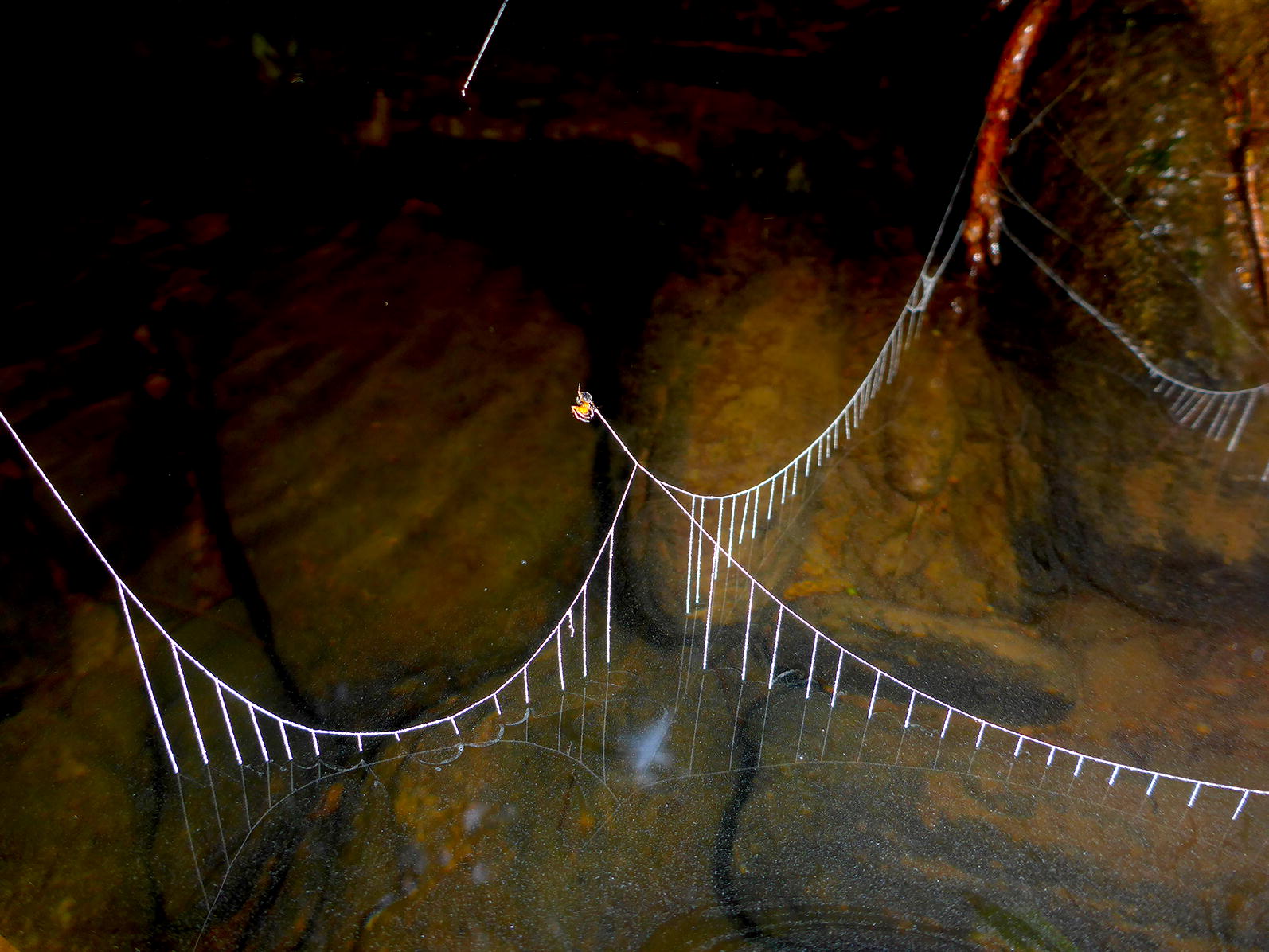
Winner: small bridges. Attribution: Darko Davor Cotoras Viedma (California Academy of Sciences, USA)

My choice as the Editor is entitled ‘Small Bridges’ and was captured by Darko Davor Cotoras Viedma of California Academy of Sciences, United States. It provides a unique perspective of a bridge created by *Wendilgarda galapagensis*, a species of spider, allowing us to appreciate a masterpiece created by a non-human architect (Fig. 8).

Cotoras explains “The species *Wendilgarda galapagensis* (Theridiosomatidae) is endemic to the remote Isla del Coco in the Eastern Tropical Pacific. This spider belongs to a genus that produces a highly specialized web. The horizontal lines are structural equivalents to the radial lines on a typical orb web. The vertical lines correspond to sticky threads for capturing prey; equivalent to the spiral line in other spiders’ webs. These delicate structures resemble hanging bridges across the still waters of a small ravine.”

## Highly commended

### Behavioral ecology and interactions with the environment

Amongst the strongest entries for the 2018 competition, were those that showcased some of the ways in which animals interact with their environment and protect themselves from danger.

This is illustrated beautifully by Arnaud Badiane’s image of a blue-tongued skink lizard. When threatened, the lizard sticks out an enormous, curled tongue that is the most brilliant shade of blue. Remarkably, the tongue is also ultraviolet and is used to scare off predators (Additional file 1).

In another image, by David Costantini, a mother raccoon can be seen walking close to her baby. Raccoons are generally solitary animals, but mothers will stay with their young for many months, feeding them and teaching them how to survive on their own (Additional file 2).

### Biodiversity and conservation ecology

Another prevalent theme in this years competition was conservation ecology and biodiversity.

One of the submitted images, taken by Joseph Dubrovsky, showcases a species of cactus (*Pachycereus pringlei*), where a disease that causes tumorous formations is very common in some populations, (Additional file 3).

Sometimes a photo can capture behaviors that would never be noticed with the naked eye, such as a fly feeding on the moisture of a frog’s eye, as shown in an image taken by Jorge Enrique Garcia Melo (Additional file 4).

Other submissions showed animals behaving in unusual ways that were out of step with the majority of the population. Dalmatian pelicans are amongst the largest freshwater birds in the world, but their numbers have declined dramatically due to a loss of habitat and poaching. They are currently considered an endangered species. Lake Kerkini is home to a major colony of Dalmatian Pelicans, which are fed, in part, by ecotourist guides.

The photographer, Nayden Charkarov, explains the unusual behavior of the birds: “Most of these birds fight each other for the large fish on offer, but some individuals avoid confrontation and break away to instead hunt naturally for flocks of smaller fish.” Charkarovs entry shows one of the birds enjoying the fruits of his labors (Additional file 5).

### Beautiful landscapes

One of the most striking images amongst the various entries featuring landscapes shows *Espeletia*, a perennial subshrub of the sunflower family. The image was captured by Marta Kolanowska while walking through the Colombian Paramo (Additional file 6).

## Conclusions

As in previous years, the quality and variety of the images submitted to the 2018 BMC Ecology image competition was excellent. We thank everyone who participated in this year’s competition and congratulate the winning photographers. We hope that our readers enjoy the collection of images as much as we do, and we look forward to the 2019 competition!

## Additional files

**Additional file 1.** “When threatened, bluetongue skinks (*Tiliqua scincoides*) open their jaws to reveal a most unusual tongue. What is remarkable is not just that this tongue is enormous, or that it flicks and curls as it protrudes from the lizard’s mouth, but that this tongue is the most brilliant shade of blue. Or is it? In fact, recent studies have shown that this tongue is actually ultraviolet and is used to scare off predators”. Attribution: Arnaud Badiane (iEES-Paris, Sorbonne University, Paris, France).

**Additional file 2.** “This photo made in Vancouver (Canada) shows a raccoon (*Procyon lotor*) mum with her baby. Although raccoons live generally solitary, mothers and young stay together for many months during which mothers protect and feed their babies and teach them how to survive on their own”. Attribution: David Costantini (Muséum National d’Histoire Naturelle, Paris, France).

**Additional file 3.** “*Pachycereus pringlei* (cardon) cacti a dominant species in the Sonoran Desert. We have documented that a tumorous formations are common in some populations (Dubrovsky and Leon de la Luz, 1996). Now, 22 years later, I visited these populations and found that this disease is still prolific in the same populations”. Attribution: Joseph Dubrovsky (Departamento de Biologia Molecular de Plantas, Instituto de Biotecnología, Universidad Nacional Autónoma de México).

**Additional file 4.** A small fly feeding on the moisture of a frog’s eye (*Pithecopus hypochondrialis*). Llanos of Colombia. Attribution: Jorge Enrique García Melo (Grupo de Investigación en Zoología, Universidad del Tolima).

**Additional file 5.** “Most pelicans in Lake Kerkini, Greece fight each other for large fish distributed by ecotourism guides. Single individuals resist this trend. They hunt flocks of small fish in a natural manner, while avoiding most attention and competition to get modest but reliable rewards. For the fish, the high vantage point is usually their last one”. Attribution: Nayden Chakarov (Department of Animal Behaviour, Bielefeld University, Germany).

**Additional file 6.** Walking within Espeletia in Colombian paramo. Attribution: Marta Kolanowska (University of Lodz, Poland).
