# Supplementary figures and images for: BMC ecology image competition 2018: the winning images
Source: BMC Ecol. 2019 Mar 8;19:11. doi: 10.1186/s12898-019-0226-z (PMC6407182; doi:10.1186/s12898-019-0226-z)

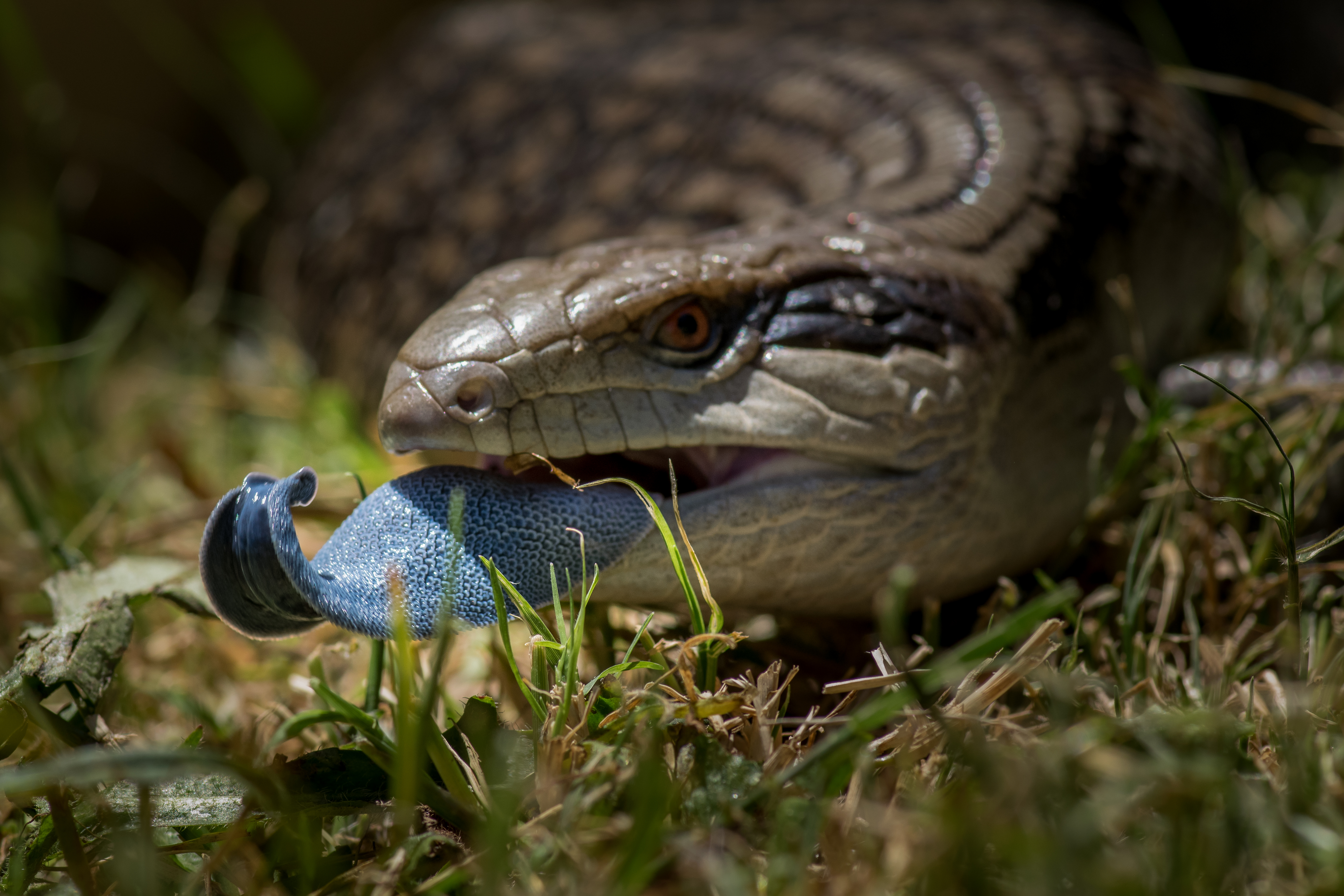

Supplement: Supplementary file 1 — Additional file 1. “When threatened, bluetongue skinks (Tiliqua scincoides) open their jaws to reveal a most unusual tongue. What is remarkable is not just that this tongue is enormous, or that it flicks and curls as it protrudes from the lizard’s mouth, but that this tongue is the most brilliant shade of blue. Or is it? In fact, recent studies have shown that this tongue is actually ultraviolet and is used to scare off predators”. Attribution: Arnaud Badiane (iEES-Paris, Sorbonne University, Paris, France). [file 12898_2019_226_MOESM1_ESM.jpg]

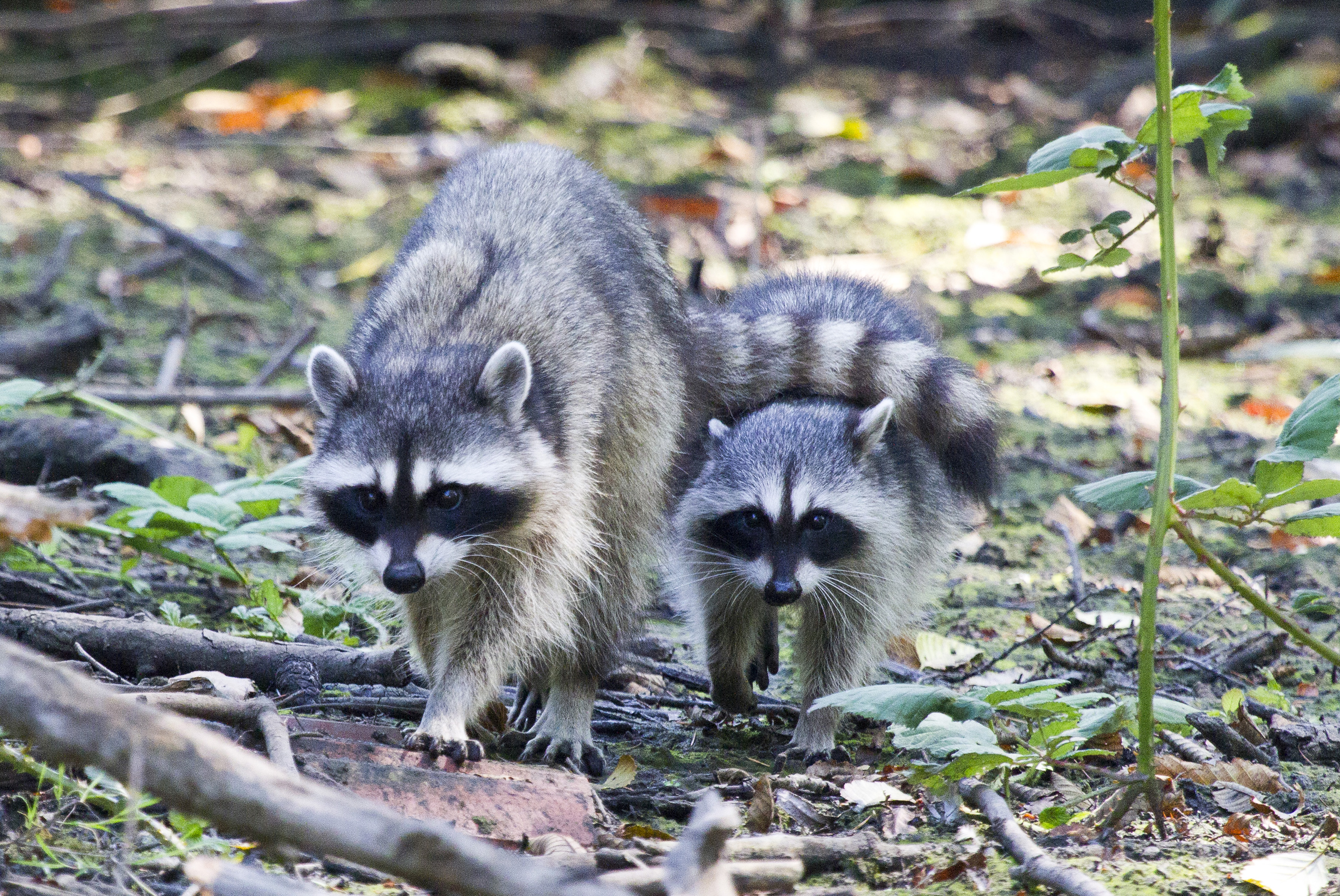

Supplement: Supplementary file 2 — Additional file 2. “This photo made in Vancouver (Canada) shows a raccoon (Procyon lotor) mum with her baby. Although raccoons live generally solitary, mothers and young stay together for many months during which mothers protect and feed their babies and teach them how to survive on their own”. Attribution: David Costantini (Muséum National d’Histoire Naturelle, Paris, France). [file 12898_2019_226_MOESM2_ESM.jpg]

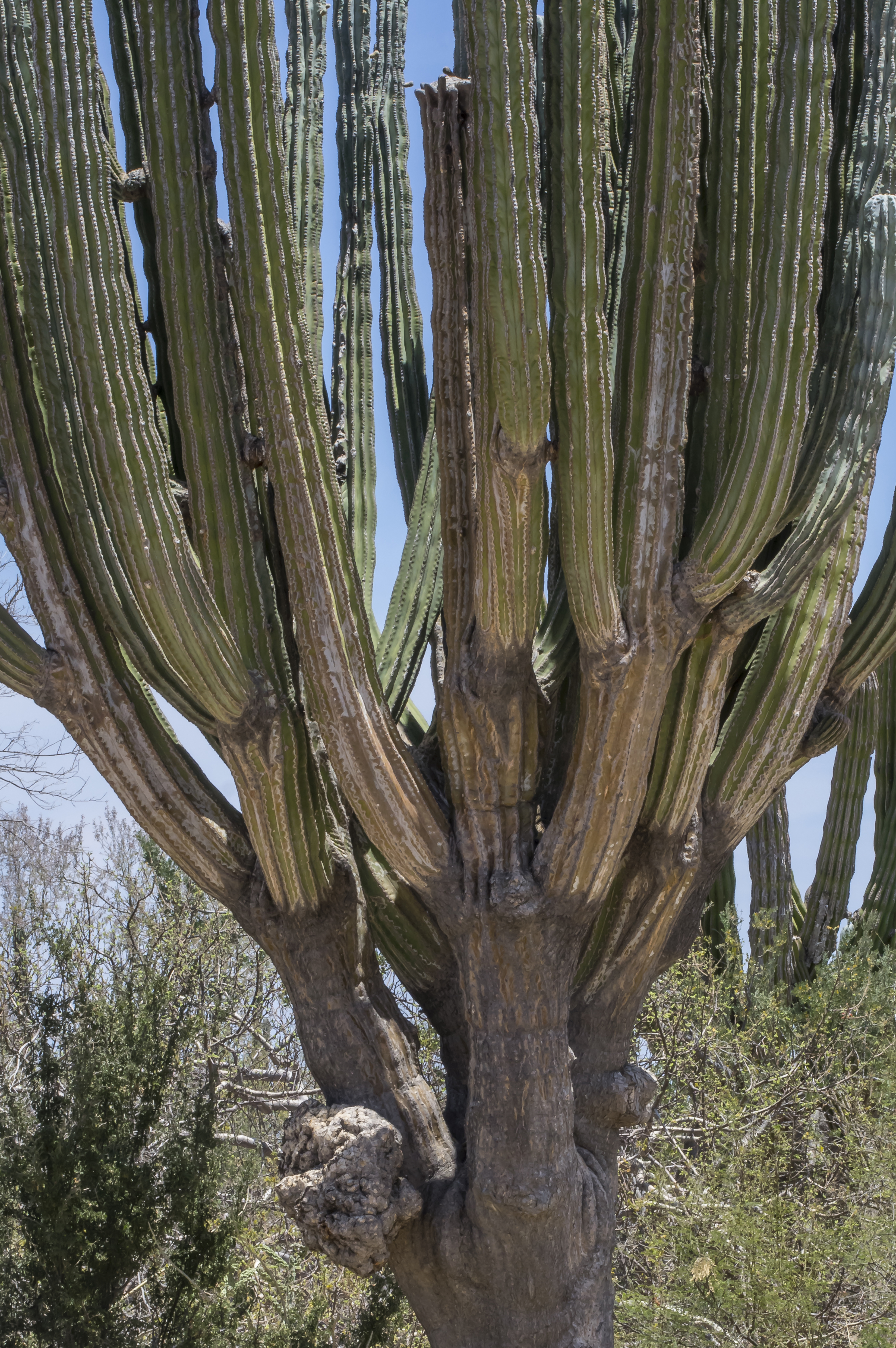

Supplement: Supplementary file 3 — Additional file 3. “Pachycereus pringlei (cardon) cacti a dominant species in the Sonoran Desert. We have documented that a tumorous formations are common in some populations (Dubrovsky and Leon de la Luz, 1996). Now, 22 years later, I visited these populations and found that this disease is still prolific in the same populations”. Attribution: Joseph Dubrovsky (Departamento de Biologia Molecular de Plantas, Instituto de Biotecnología, Universidad Nacional Autónoma de México). [file 12898_2019_226_MOESM3_ESM.jpg]

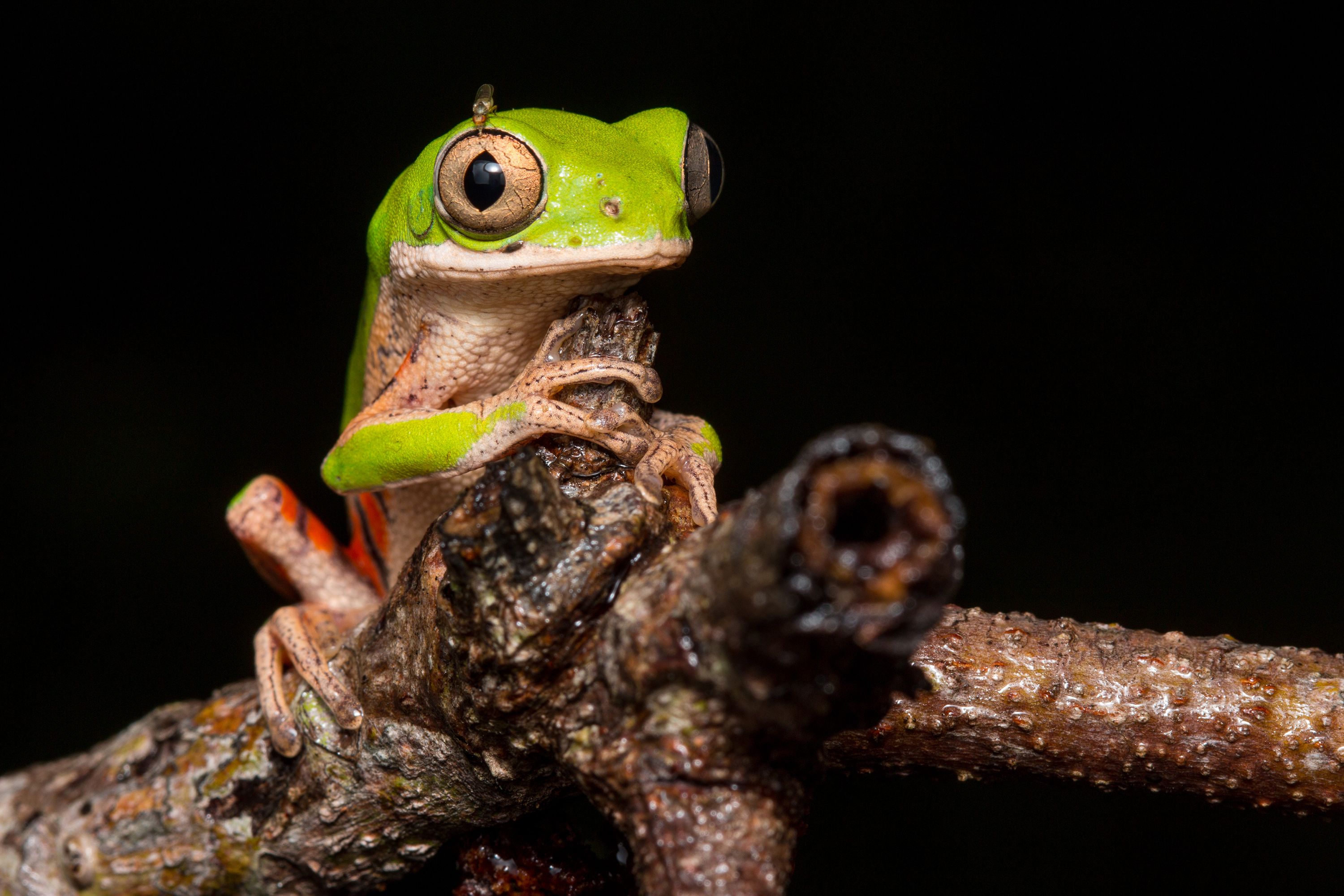

Supplement: Supplementary file 4 — Additional file 4. A small fly feeding on the moisture of a frog’s eye (Pithecopus hypochondrialis). Llanos of Colombia. Attribution: Jorge Enrique García Melo (Grupo de Investigación en Zoología, Universidad del Tolima). [file 12898_2019_226_MOESM4_ESM.jpg]

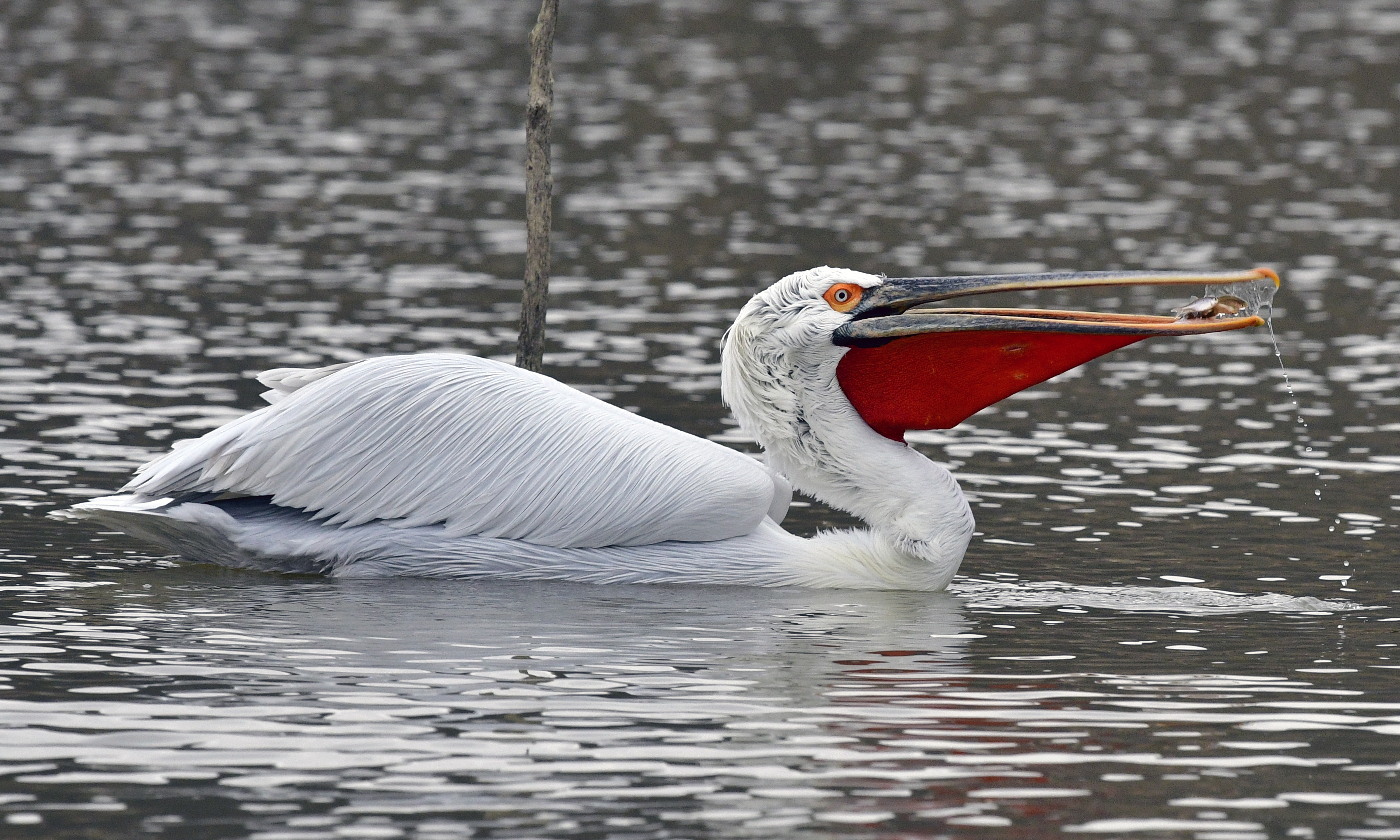

Supplement: Supplementary file 5 — Additional file 5. “Most pelicans in Lake Kerkini, Greece fight each other for large fish distributed by ecotourism guides. Single individuals resist this trend. They hunt flocks of small fish in a natural manner, while avoiding most attention and competition to get modest but reliable rewards. For the fish, the high vantage point is usually their last one”. Attribution: Nayden Chakarov (Department of Animal Behaviour, Bielefeld University, Germany). [file 12898_2019_226_MOESM5_ESM.jpg]

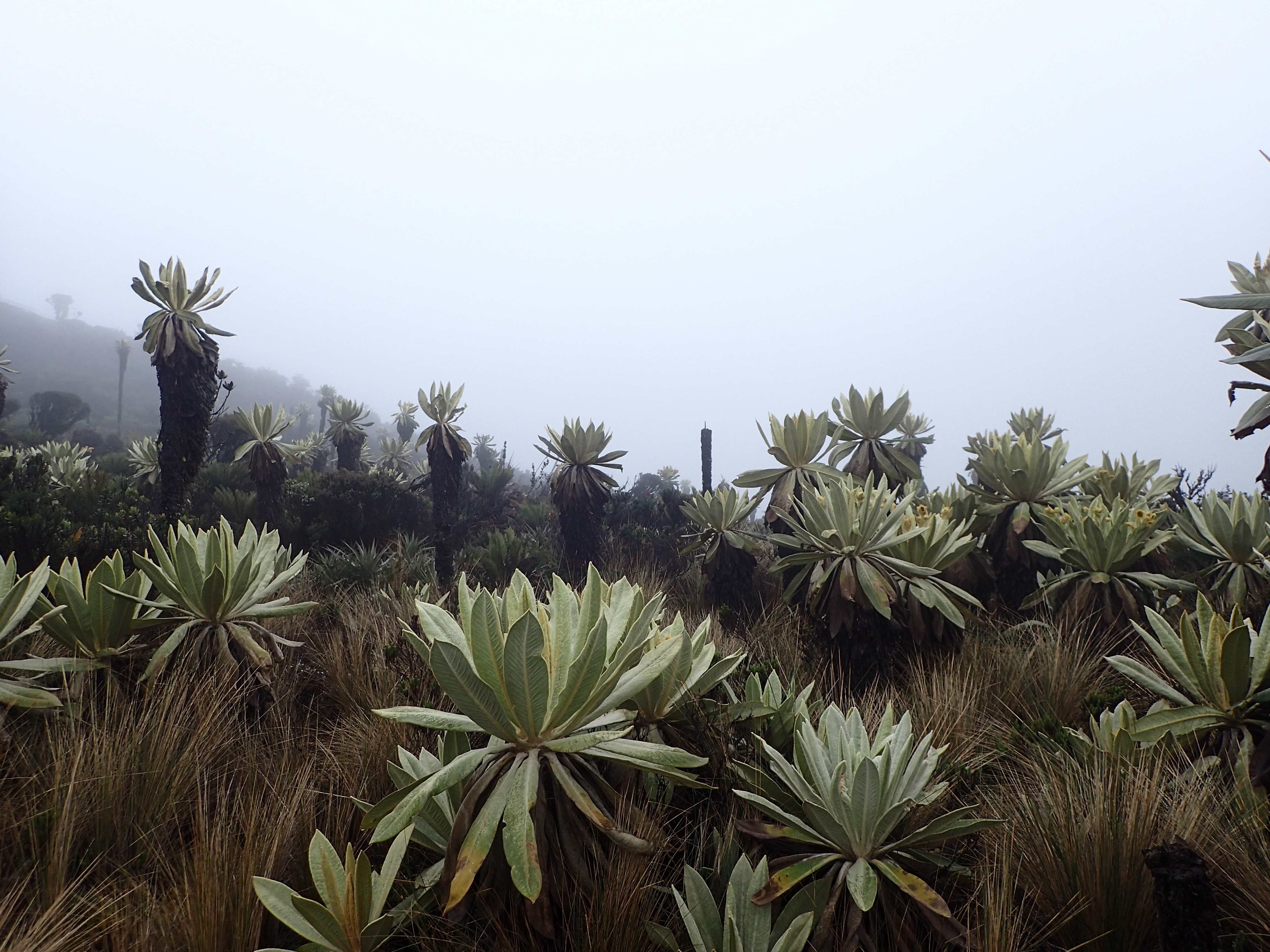

Supplement: Supplementary file 6 — Additional file 6. Walking within Espeletia in Colombian paramo. Attribution: Marta Kolanowska (University of Lodz, Poland). [file 12898_2019_226_MOESM6_ESM.jpg]
